# Differences in *Staphylococcus aureus* nasal carriage and molecular characteristics among community residents and healthcare workers at Sun Yat-Sen University, Guangzhou, Southern China

**DOI:** 10.1186/s12879-015-1032-7

**Published:** 2015-07-30

**Authors:** Baiji Chen, Xinlu Dai, Bo He, Kunyi Pan, Hongyu Li, Xiaoqiang Liu, Yunwen Bao, Weisi Lao, Xiquan Wu, Yandan Yao, Songyin Huang

**Affiliations:** Department of Laboratory, Guangdong Provincial Key Laboratory of Malignant Tumor Epigenetics and Gene Regulation, Sun Yat-Sen Memorial Hospital, Sun Yat-Sen University, Guangzhou, 510120 China; Department of Anesthesiology, Sun Yat-Sen Memorial Hospital, Sun Yat-Sen University, Guangzhou, 510120 China; Breast Tumor Center, Sun Yat-Sen Memorial Hospital, Sun Yat-Sen University, Guangzhou, Guangdong 510120 China

**Keywords:** *Staphylococcus aureus*, Nasal carriage, Antimicrobial susceptibility, Healthcare worker, Community resident, Infection control

## Abstract

**Background:**

The pathogenic potential and commensal nature of *Staphylococcus aureus* allows for easy transmission both within and outside of the hospital environment, and nasal carriage may be responsible for some serious infections. This study aimed to determine the molecular and epidemiological characteristics of nasal colonization by *S. aureus* in community residents (CR) and healthcare workers (HW) at Sun Yat-Sen University, Guangzhou, China.

**Methods:**

A total of 589 volunteers, both CR (*n* = 297) and HW (*n* = 292), were recruited. Each subject completed a questionnaire, and specimens were obtained from the anterior nares for *S. aureus* screening. Genotypic analysis included pulsed-field gel electrophoresis (PFGE), multilocus sequence typing (MLST), staphylococcal cassette chromosome *mec* (SCC*mec*) typing, and virulence gene detection.

**Results:**

A total of 138 *S. aureus* isolates were recovered from separate subjects (23.4 %, 138/589), with four isolates showing methicillin resistance (0.7 %, 4/589). The prevalence of *S. aureus* carriage was 25.3 % (75/297) in CR and 21.6 % (63/292) in HW. Methicillin-resistant *S. aureus* (MRSA) were isolated from one CR (0.3 %, 1/297) and three HW (1.0 %, 3/292). The most common risk factors for *S. aureus* carriage in CR were being male, age ≤ 30 years, and nasal cavity cleaning habits. Having a household member in the healthcare profession was associated with increased risk among HW. Sequence type (ST)188 and ST59 were the most prevalent among the 20 observed STs, accounting for 14.6 % and 12.2 % of all isolates, respectively. The four MRSA isolates presented four different STs, with one isolate carrying a type IVa SCC*mec* element and the other three isolates containing type III SCC*mec*. PFGE analysis grouped the 129 isolates into 23 pulsotypes, with profiles A, N, E, L, and O the most prevalent. The Panton-Valentine leucocidin gene (*pvl*) was identified in two of the 138 isolates, while 57.5 % of isolates carried both the *Staphylococcus aureus* enterotoxin A (*sea*) and enterotoxin B (*seb*) genes.

**Conclusions:**

These data indicate a low prevalence of nasal MRSA carriage but evidence of molecular heterogeneity among *S. aureus* isolates from CR and HW at Sun Yat-Sen University, Guangzhou. Differences in epidemiological and molecular characteristics of *S. aureus* between CR and HW populations may be useful for the understanding and prevention of *S. aureus* infection.

**Electronic supplementary material:**

The online version of this article (doi:10.1186/s12879-015-1032-7) contains supplementary material, which is available to authorized users.

## Background

*Staphylococcus aureus* colonizes various body sites, but the anterior nares are the most consistent site of colonization [1]. As a medically important pathogen, colonization is a strong risk factor for subsequent infection; however, most people colonized with *S. aureus* do not develop clinical disease [2]. Why some individuals are apparently resistant to colonization, and thus at lower risk of infection, remains unknown. Risk factors for colonization include young age, being male, underlying comorbidities, hospitalization, and exposure to livestock [2–4]. Determination of the prevalence of *S. aureus* nasal carriage in healthy populations, as well as resistance gene profiling and molecular typing of nasal *S. aureus* isolates, is beneficial for identifying risk factors associated with *S. aureus* infection [5, 6]. Molecular epidemiological studies have shown that a limited number of methicillin-resistant *S. aureus* (MRSA) strains have spread by clonal dissemination between different hospitals, cities, countries, and even continents, and now cause healthcare-associated MRSA (HA-MRSA) infections worldwide [7, 8]. Like HA-MRSA, successful clones of community-associated MRSA (CA-MRSA) are usually associated with specific geographical locations [9]. Molecular typing of *S. aureus* is also helpful for supporting infection control measures, investigating suspected outbreaks, and preventing nosocomial transmission [7, 10]. Data on *S. aureus* nasal carriage in the community largely comes from developed countries [3, 5, 6], and reports from China are very limited. Previous studies revealed 15.4–23.1 % *S. aureus* nasal carriage in Chinese medical students from different regions, of which, 3.0–9.4 % were MRSA [11, 12]. Another study revealed a similar nasal carriage rate (16.5 %) and low prevalence of MRSA colonization (0.3 %) in 2448 healthy people from Beijing and Harbin [13]. In contrast, a relatively high prevalence of MRSA colonization was found (11.6 %) in a cohort of healthy children aged ≤ 14 years in community settings in Taiwan over a 5-year period [14].

The epidemiology of *S. aureus* among the general population in Guangzhou, including community residents (CR) and healthcare workers (HW), has not been studied. Therefore, we sought to determine the prevalence, antimicrobial resistance profiles, toxin gene expression, and molecular characteristics of nasal *S. aureus* isolates from healthy individuals at Sun Yat-Sen University, Guangzhou, China.

## Methods

### Study design and population

This cross-sectional study was conducted between October 2013 and March 2014 in two communities from the campuses of Sun Yat-Sen University (South Campus and North Campus) and Sun Yat-Sen Memorial Hospital, Sun Yat-Sen University, in Guangzhou, Southern China. Sun Yat-Sen University is a comprehensive multi-disciplinary university covering a total area of 5.97 km^2^, and has four campuses: Guangzhou South Campus, Guangzhou North Campus, Guangzhou East Campus, and Zhuhai Campus. It has about 82,384 students studying on-campus in Guangzhou. Sun Yat-Sen Memorial Hospital is also known as the Second Affiliated Hospital of Sun Yat-Sen University. With more than 4,200 staff and 2,200 inpatient beds available, the hospital performs over 50,000 inpatient operations, discharges about 80,000 inpatients, and handles more than 3 million outpatient visits annually. The three communities are located in downtown Guangzhou, Southern China.

A total of 589 volunteers aged 11–78 years participated in this study: 297 CR were randomly recruited from the South and North campuses of Sun Yat-Sen University (middle school students, undergraduates, teachers, salesclerks, and retirees), and 292 HW (doctors, nursing staff, clinical laboratory staff, administration clerks, and cleaners) were randomly recruited from Sun Yat-Sen Memorial Hospital. All volunteers, parents, or guardians signed informed consent documents approving the use of their samples for research purposes, and the study was approved by the Ethics Committee of Sun Yat-Sen Memorial Hospital.

Using a standardized questionnaire, information regarding the following demographic data and risk factors were obtained from each participant: age, gender, profession, use of antibiotics in the last 4 weeks, nasal cavity cleaning habits, hospitalization in the last 12 months, underlying disease, and family members working in a healthcare profession. The questionnaires were sent out, and the subjects were required to independently finish the questionnaires. The completed questionnaires were collected at the time of sampling. During data collation, 28 surveys were eliminated because responses were incomplete. Therefore, a total of 589 complete surveys were obtained, with an efficiency rate of 95.5 %. Nasal sampling was carried out independently by five well-trained nurses and technicians. A STROBE-Checklist for this study was provided (Additional file 1).

### Isolation and identification of nasal *S. aureus* isolates

The nasal sampling procedure for the screening of *S. aureus* nasal carriage was standardized to ensure accurate sample collection and completion of microbiological procedures. For each subject, a nasal swab specimen was collected from the anterior nares using Copan eSwab Liquid Amies preservation medium (eSwab Collection and Preservation System, Copan Italia, Brescia, Italy). Samples were collected by rotating a sterile cotton swab five times in both anterior nares. These swabs were then transported at room temperature and processed within 4 h. Samples were first cultured on blood agar plates for 24 h at 35 °C. Gram-positive, β-hemolytic, coagulase-positive isolates were confirmed as *S. aureus* using a Vitek®2 microbial identification system (bioMérieux, Marcy l’Etoile, France) according to the manufacturer’s instructions.

### Antibiotic susceptibility testing

Antibiotics used for susceptibility testing included penicillin, erythromycin, clindamycin, cefuroxime, ceftriaxone, cefotaxime, cefoxitin, gentamicin, rifampicin, imipenem, tetracycline, quinupristin/dalfopristin, teicoplanin, vancomycin, ciprofloxacin, trimethoprim/sulfamethoxazole, and levofloxacin. Susceptibilities were determined using the disk diffusion method in accordance with the performance standards for antimicrobial susceptibility testing, 23rd informational supplement (M100-S23), recommended by the Clinical and Laboratory Standards Institute (CLSI; http://clsi.org). All disks were obtained from Oxoid Ltd (Oxoid, Basingstoke, England), and *S. aureus* ATCC 25923 was used as the quality control strain. Multidrug resistance was arbitrarily defined as resistance of methicillin-sensitive *S. aureus* (MSSA) to three or more distinct antimicrobial classes. MRSA strains isolated in this study were included in the multidrug-resistant (MDR) category irrespective of their susceptibility profiles.

### Detection of staphylococcal toxin genes

All of the *S. aureus* isolates were screened for the presence of the Panton-Valentine leukocidin (*pvl*) and the *Staphylococcus aureus* enterotoxin A (*sea*) and enterotoxin B (*seb*) genes by PCR using primers and methods described previously [15, 16].

### Staphyloccoccal cassette chromosome *mec* (SCC*mec*) typing

SCC*mec* typing of MRSA isolates was performed using eight unique pairs of primers specific for SCC*mec* types and subtypes I, II, III, IVa, IVb, IVc, IVd, and V, as described previously [12, 17]. Positive control strains for SCC*mec* types I (NCTC 10442), II (N315), III (85/2082), and IVa (JCSC 4744), were kindly provided by Dr. Fangyou Yu of the Department of Laboratory Medicine, the First Affiliated Hospital of Wenzhou Medical College.

### Multilocus sequence typing (MLST)

Forty-one *S. aureus* isolates (including 12 isolates from CR in the South Campus, 11 isolates from CR in the North Campus, and 18 isolates from HW) were randomly selected and investigated by MLST. MLST was performed as described previously [18], and the sequences of the PCR products were compared with an MLST database (http://saureus.mlst.net). eBURST software was used to cluster related sequence types (STs), which were defined as clonal complexes (CCs) (http://eburst.mlst.net/v3/enter_data/single). A neighbor-joining tree was constructed from the sequence data using MEGA version 5 [10]. STs that grouped together with ≥70 % bootstrap support were considered part of the same CC.

### Pulsed-field gel electrophoresis (PFGE)

The clonal relationships of all 138 isolates were assessed by PFGE using *Sma*I as previously described [19]. The PFGE types were defined according to the criteria of Tenover et al. [20]. The isolates with > 75 % similarity were clustered in patterns. The results were also analyzed using BioNumerics version 5.01 statistical software, and dendrograms were generated according to a simple matching coefficient and the unweighted pair group method with the arithmetic mean (UPGMA) algorithm.

### Statistical analysis

In descriptive statistics, frequency and proportions were calculated for categorical variables. Categorical variables were compared using the chi-square test or the Fisher exact test. The only continuous variable, age, was transformed into a categorical variable using the quartiles of the frequency distribution (≤20, > 20–30, > 31–50, > 50 years). Odds ratios (OR), 95 % confidence intervals (CI), and P-values were calculated. Possible determinants for *S. aureus* nasal carriage were first checked through univariable logistic regression analysis. We applied multiple logistic regressions by stepwise backward selection of variables with biological plausibility and a significance level < 0.10 for entry into the model. All statistical tests were considered significant with a P-value < 0.05. Data were analyzed using IBM SPSS Statistics for Windows version 18. All susceptibility data and molecular test results were analyzed using WHONET software, version 5.6.

## Results

### Nasal *S. aureus* carriage

Demographic characteristics of the study population are shown in Table 1. The median age of the participating volunteers was 26.5 years (range 11–78 years), and 240 (41.6 %) were male. There were some significant differences between the demographic profiles of the CR and HW groups, including age distributions (*P* < 0.001) and gender (*P* = 0.012). Distributions of *S. aureus* carriers and non-carriers stratified by population characteristics are shown in Tables 2 and 3. *S. aureus* was detected in the nasal swabs of 138 participants (23.4 %, 138/589). The overall prevalence of *S. aureus* carriage was 25.3 % (75/297) in CR and 21.6 % (63/292) in HW (OR = 1.20, 95 % CI: 0.81–1.76). The corresponding age-specific rates were 27.9 % and 22.0 %, respectively, for those aged 20–30 years (OR = 0.99, 95 % CI: 0.88–1.10), and 17.0 % and 22.3 %, respectively, for those aged 30–50 years (OR = 1.00, 95 % CI: 0.93–1.07). The corresponding sex-specific rates were 18.6 % and 21.3 %, respectively, for female participants (OR = 0.81, 95 % CI: 0.48–1.38), and 33.1 % and 22.5 %, respectively, for males (OR = 1.74, 95 % CI: 0.96–3.15). In multivariable analysis, HW status was not associated with *S. aureus* nasal carriage in the total population (Table 2).

**Table 1:** Demographic characteristics of the CR and HW groups from Sun Yat-Sen University, Guangzhou, Southern China

Characteristic		Demographic characteristics, n (%)		
	Level	CR	HW	P-value
		(*n* = 297)	(*n* = 292)	
Gender	Male	136(56.7)	104(43.3)	0.012
	Female	161(46.1)	188(53.8)	
Age, years	≤20	126(95.5)	6(4.5)	<0.001
	>20–30	68(28.2)	173(71.8)	
	>30–50	47(31.3)	103(68.7)	
	>50	56(84.8)	10(15.2)	
Use of antibiotics in the last 4 weeks	Yes	20(47.6)	22(52.4)	0.723
	No	277(50.6)	270(40.4)	
^b^Nasal cavity cleaning habits	Frequently	100(44.6)	124(55.4)	0.028
	Occasionally	197(53.9)	168(46.1)	
Prior hospitalization in the last 12 months	Yes	11(44.0)	14(56.0)	0.512
	No	286(50.7)	278(40.3)	
^a^Underlying disease	Yes	30(75.0)	10(25.0)	<0.001
	No	252(47.2)	282(52.8)	
Any family member a HW	Yes	52(40.0)	79(60.0)	<0.001
	No	295(68.6)	135(31.4)	

^a^Underlying disease: hypertension, diabetes, chronic rhinitis, urticaria, hyperthyroidism

^b^Nasal cavity cleaning habits: frequently, at least once per day; occasionally, less than once per day

**Table 2:** Estimated risk of *Staphylococcus aureus* nasal carriage by healthcare worker status

Characteristic		Nasal carriage of *S.aureus*, n (%)					
		Carriers	Noncarriers	Univariate		Multivariate logistic	
				P-value	OR(95 % CI)	P-value	OR(95 % CI)
Both genders							
	CR	75(25.3)	222(74.7)	0.363	1.20(0.81–1.76)		
	HW	63(21.6)	229(78.4)				
Age, years	>20–30						
	CR	17(27.9)	51(72.1)	0.816	0.99(0.88–1.10)		
	HW	38(22.0)	135(78.0)				
	>30–50						
	CR	8(17.0)	39(83.0)	0.989	1.00(0.93–1.07)		
	HW	23(22.3)	80(77.7)				
Female							
	CR	30(18.6)	131(81.4)	0.436	0.81(0.48–1.38)		
	HW	40(21.3)	148(78.7)				
Male							
	CR	45(33.1)	91(66.9)	0.069	1.74(0.96–3.15)	0.155	1.56(0.85–2.88)
	HW	23(22.5)	81(77.5)				

*CR* community residents, *HW* healthcare workers, *OR* odds ratio, *CI* confidence interval

**Table 3:** Univariate and multivariate analysis of risk factors associated with *Staphylococcus aureus* nasal carriage between CR and HW at Sun Yat-Sen University, Guangzhou, Southern China

Characteristic		CR (*n* = 297), n (%)						HW (*n* = 292), n (%)					
		Carriers	Noncarriers	Univariate		Multivariate logistic		Carriers	Noncarriers	Univariate		Multivariate logistic	
		(*n* = 75)	(*n* = 222)	P-value	OR(95 % CI)	P-value	OR(95 % CI)	(*n* = 63)	(*n* = 229)	P-value	OR(95 % CI)	P-value	OR(95 % CI)
Gender	Male	45(33.1)	91(66.9)	0.004	2.16(1.27–3.68)	0.005	2.15(1.27–3.68)	23(22.5)	81(77.5)	0.871	1.05(0.59–1.88)		
	Female	30(18.6)	131(81.4)					40(21.3)	148(78.7)				
Age, years	≤20	43(34.1)	83(65.9)	0.003	0.23(0.09–0.58)	0.004	3.88(1.54–9.79)	1(16.7)	5(83.3)	1.089	0.56(0.03–10.93)		
	>20–30	18(26.5)	50(73.5)	0.032	0.33(0.12–0.91)	0.047	2.78(1.01–7.62)	38(22.0)	135(78.0)	0.619	0.40(0.05–3.21)		
	>30–50	8(17.0)	39(83.0)	0.356	0.59(0.19–1.83)	0.356	1.71(0.55–5.34)	23(22.3)	80(77.7)	0.61	0.39(0.05–3.21)		
	>50	6(10.7)	50(89.3)		1		1	1(10.0)	9(90.0)		1		
Use of antibiotics in the last 4 weeks	Yes	4(20.0)	16(80.0)	0.577	0.73(0.24–2.24)			3(13.6)	19(86.4)	0.356	0.55(0.16–1.93)		
	No	71(25.6)	206(74.4)					60(22.2)	210(77.8)				
^b^Nasal cavity cleaning habits	Frequently	13(13.0)	87(87.0)	<0.001	0.33(0.17–0.63)	<0.001	6.90(3.62–13.17)	23(18.5)	101(81.5)	0.282	0.73(0.41–1.30)		
	Occasionally	62(31.5)	135(68.5)					40(23.8)	128(76.2)				
Prior hospitalization in the last 12 months	Yes	4(36.4)	7(63.6)	0.393	1.73(0.49–6.09)			3(21.4)	11(78.6)	1.001	0.99(0.27–3.67)		
	No	71(24.8)	215(75.2)					60(21.6)	218(78.4)				
^a^Underlying disease	Yes	11(36.7)	19(63.3)	0.134	1.84(0.83–4.06)			2(20.0)	8(80.0)	0.902	0.906(0.19–4.38)		
	No	64(24.0)	203(76.0)					61(21.6)	221(78.4)				
Any family membera HW	Yes	13(25.0)	39(75.0)	0.963	0.984(0.49–1.96)			13(16.5)	66(83.5)	<0.001	0.34(0.17–0.67)	0.002	2.97(1.50–5.95)
	No	62(25.3)	183(74.7)					50(37.0)	85(63.0)				

^a^Underlying disease: hypertension, diabetes, chronic rhinitis, urticaria, hyperthyroidism

^b^Nasal cavity cleaning habits: frequently, at least once per day; occasionally, less than once per day

*CR* community residents, *HW* healthcare workers, *OR* odds ratio, *CI* confidence interval

Independent risk factors for *S. aureus* carriage differed between the CR and HW groups. Variables associated with *S. aureus* carriage in the univariate analysis are shown in Table 3. Statistically significant factors for the CR group included: male (OR = 2.16, 95 % CI: 1.27–3.68), age ≤ 30 years (≤ 20 years, OR = 0.23, 95 % CI: 0.09–0.58; 20–30 years, OR = 0.33, 95 % CI: 0.12–0.91) and nasal cavity cleaning habits (regular cleaning of the nasal cavity, OR = 0.33, 95 % CI: 0.17–0.63). In multiple logistic regression analysis, nasal carriage of *S. aureus* was also significantly associated with male (OR = 2.15, 95 % CI: 1.27–3.68), age ≤ 20 years (OR = 3.88, 95 % CI: 1.54–9.79), age 20–30 years (OR = 2.78, 95 % CI: 1.01–7.62), and regular cleaning of the nasal cavity (OR = 6.90, 95 % CI: 3.62–13.17). For the HW group, no significant association was observed between nasal *S. aureus* carriage and sex, age, use of antibiotics in the 4 weeks prior to sampling, nasal cavity cleaning habits, hospitalization in the 12 months prior to sampling, and underlying disease. However, having family members in the healthcare profession was associated with increased risk (OR = 2.97, 95 % CI: 1.50–5.95) (Table 3).

### Antimicrobial susceptibility

**Fig. 1:**
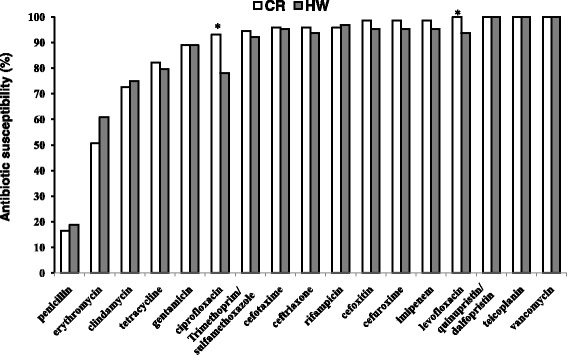
Antibiotic susceptibility profiles of 138 *Staphylococcus aureus* isolates. HW vs. CR, *P < 0.05

Among the 138 *S. aureus* isolates, 114 (82.5 %) were resistant to penicillin and 55 (39.5 %) to erythromycin. Rates of resistance to clindamycin and tetracycline were 14.6 % and 18.9 %, respectively, but were < 10 % for cefuroxime, ceftriaxone, cefotaxime, cefoxitin, gentamicin, trimethoprim/sulfamethoxazole, rifampicin, and imipenem (Fig. 1, Additional file 2: Table S2). All isolates were susceptible to teicoplanin, quinupristin/dalfopristin, and vancomycin. The level of resistance to quinolones (ciprofloxacin and levofloxacin) was significantly higher in HW compared with CR (4.7 % vs. 0, *P* < 0.001). Moreover, erythromycin resistance was less prevalent among *S. aureus* isolates from HW compared with those from CR (34.4 % vs. 43.8 %, *P* = 0.323), whereas there were no detectable differences for the remaining antimicrobials (Fig. 1, Additional file 2: Table S2).

Nine (6.5 %, 9/138) isolates were MDR, with penicillin-erythromycin-tetracycline (*n* = 6) being the predominant resistance profile (Additional file 3: Figure S2). Only four isolates (2.9 %, 4/138) were resistant to cefoxitin, and were confirmed to be MRSA by *mecA* PCR screening. All MRSA isolates were resistant to erythromycin and clindamycin. Only one MRSA isolate was resistant to erythromycin, clindamycin, tetracycline, and gentamycin. The subject was a 17-year-old female middle school student with chronic rhinitis who had taken antithyroid drugs. She therefore had a history of exposure to a hospital environment within the 12 months prior to sampling (Table 4).

**Table 4:** Molecular typing and characteristics of four MRSA isolates

CC	MLST	SCC*mec*	PFGE profile	*pvl*	*sea*	*seb*	Resistance profile	Source		
								Department	Gender	Age, years
CC8	ST8	III	D	−	−	+	ERY,CLI,TCY,GEN	South Campus	Female	17
CC121	ST120	III	T	−	+	+	ERY,CLI,TCY	Operating room	Female	34
CC10	ST10	III	G	−	−	−	ERY,CLI	Operating room	Female	29
CC59	ST59	IVa	C	−	−	+	ERY,CLI	Emergency department	Female	33

CC, clonal complex; MLST, multilocus sequence typing; SCC*mec*, staphylococcal cassette chromosome *mec*; PFGE, pulsed-field gel electrophoresis; *Pvl*, Panton-Valentine leucocidin gene; *sea, Staphylococcus aureus* enterotoxin A gene; *seb, Staphylococcus aureus* enterotoxin B gene; ERY, erythromycin; CLI, clindamycin; GEN, gentamycin; TCY, tetracycline

−, negative; +, positive

### Expression of virulence factors

**Fig. 2:**
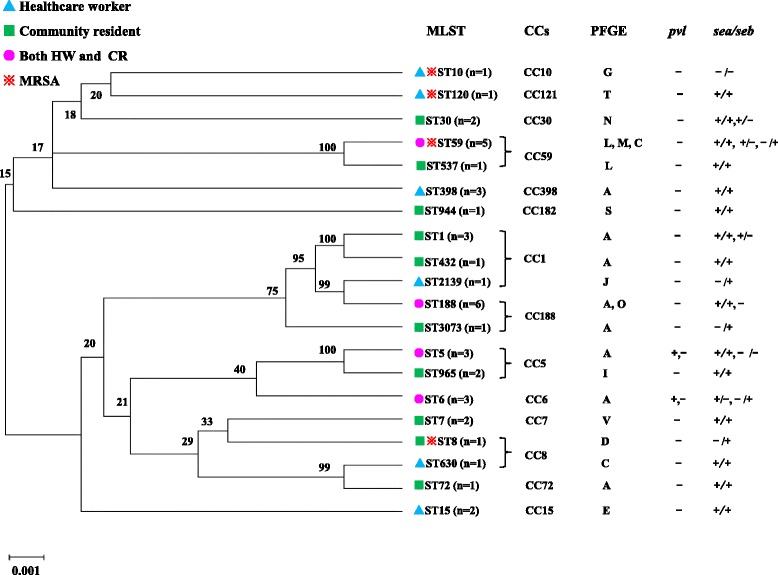
Phylogenetic relationships of 41 *Staphylococcus aureus* strains isolated from healthcare workers (HW) and community residents (CR) based on multilocus sequence typing (MLST) data. The neighbor joining tree was based on the distance matrix of pair-wise differences between sequence types (STs), determined using the *S. aureus* MLST database (http://saureus.mlst.net/), implemented in MEGA v5.1 using Kimura-2-parameter distances. The relationships shown were based on 1000 re-samplings for bootstrapping. Each clonal complex (CC) is composed of STs that cluster with a ≥ 70 % bootstrap confidence value. PFGE, pulsed-field gel electrophoresis;*Pvl*, Panton-Valentine leucocidin gene; *sea, Staphylococcus aureus* enterotoxin A gene; *seb, Staphylococcus aureus* enterotoxin B gene; −, negative; +, positive

The *pvl* gene was detected in two *S. aureus* isolates (1.4 %, 2/138) (Fig. 2, Additional file 4: Figure S1B), which were confirmed as ST5 and ST6 isolates from HW. Among all *S. aureus* isolates, 57.5 % carried both the *sea* and *seb* genes, while only 17.5 % carried the *sea* gene and 20.0 % carried the *seb* gene (Additional file 2: Table S3, Additional file 4: Figure S1C and S1D). The distribution of *S. aureus* isolates carrying both the *sea* and *seb* genes was not significantly different between the CR and HW groups (56.5 % vs. 55.6 %, *P* = 0.325). However, a significantly higher proportion of *sea* gene-positive isolates came from the CR group compared with the HW group (26.1 % vs. 5.6 %, *P* = 0.024), whereas the *seb* gene was more common among isolates from HW (22.2 % vs. 17.4 %, *P* = 0.069) (data not shown).

### SCC*mec* typing

MRSA was detected in four female participants (0.7 %, 4/589). One isolate carried a type IVa SCC*mec* element, while the other three isolates carried type III SCC*mec*. None of the MRSA isolates carried *pvl* (Table 4, Additional file 4: Figure S1A). The ST59/SCC*mec* IVa MRSA was isolated from an emergency department nurse, while the ST120/MRSA SCC*mec* III and ST10/MRSA SCC*mec* III isolates were found in operating room staff. The ST8/SCC*mec* III MRSA was isolated from a middle school student at South Campus. The distribution of the different SCC*mec* types is shown in Table 4.

### MLST and PFGE typing

Multilocus sequence typing (MLST) of 41 *S. aureus* strains revealed 20 different sequence types. Among these, ST188 and ST59 were the most prevalent, accounting for 14.6 % and 12.2 % of all isolates, respectively. ST188 and ST59 isolates were obtained from both CR and HW, while eight MLST types (ST1, ST7, ST8, ST30, ST72, ST537, ST944, ST965, and ST3073) were restricted to CR, and seven MLST types (ST10, ST15, ST120, ST398, ST432, ST630, and ST2139) were unique to HW (Fig. 2, Additional file 2: Table S3).

Based on MLST typing, the following CCs were identified among the 41 isolates: CC188 (*n* = 7), CC59 (*n* = 6), CC5 (*n* = 5), CC1 (*n* = 5), CC6 (*n* = 3), CC398 (*n* = 3), CC7 (*n* = 2), CC30 (*n* = 2), CC15 (*n* = 2), CC72 (*n* = 1), CC121 (*n* = 1), CC10 (*n* = 1), and CC182 (*n* = 1). CC188 isolates were obtained from both CR (*n* = 4) and HW (*n* = 3), whereas CC398 isolates were more common among HW. No other significant differences were observed in the occurrence of CCs among HW and CR isolates (Fig. 2, Additional file 2: Table S3).

Among the 138 *S. aureus* isolates, PFGE grouped 129 isolates into 23 pulsotypes, while nine isolates were untypeable (Additional file 3: Figure S2). Patterns were classified from A–W, each defining a clone in accordance with the previously reported interpretive criteria [20]. The most prevalent profiles were A, N, E, L, and O (each with > 5 isolates). PFGE pattern A (49.2 %, 68/138) had 40 CR isolates (25 from South Campus and 15 from North Campus) and 28 HW isolates, while PFGE pattern N (9.4 %, 13/138) had 13 CR isolates, all from South Campus. The five isolates with PFGE pattern O (4.9 %, 5/138) were all from the HW group. The four MRSA strains belonged to PFGE patterns C, D, G, and T. The remaining twenty pulsotypes showed no particular pattern, and overall, the PFGE patterns showed no significant differences among the different specimens, genders, and age groups.

## Discussion

To our knowledge, this is the first study to provide insight into the prevalence of nasal carriage, antimicrobial susceptibility, and clonal structure of *S. aureus* and MRSA in the general population of Guangzhou, China. The findings are useful for understanding *S. aureus* nasal colonization dynamics within the population, and for designing strategies to prevent *S. aureus* infection and dissemination. *S. aureus* nasal carriage is a global phenomenon that is affected by various factors including, but not limited to, age, health, economic status, and the country of residence. In the current study, nasal carriage of *S. aureus* was identified in 23.4 % of the study population. This coincides with the recorded prevalence among healthy adults in Northern China (16.5 %), and in adults in community settings in Taiwan (22.1 %) [13, 21]. In Germany and the UK, the prevalence of *S. aureus* is reported to be 21.9 % and 28 %, respectively, in the general population [5, 6]. Carriages rates within the continental USA vary from 26 to 32 %, with local population variations [22, 23]. The low prevalence of MRSA (0.7 %) and the heterogeneity of MLST types in the current study suggest that there were no singularly expanding MRSA clones among the study population. The prevalence of MRSA is similar to that identified in a previous study (0.33 %) in North China [13], but is lower than the prevalence reported from a large population-based random sample of 3,098 adult participants in Taiwan (3.8 %) [21]. The current MRSA prevalence is also similar to the estimates in Germany (1.29 %), the UK (1.1 %), and the USA (0.8 %), taking the reported confidence intervals into account [5, 6, 23].

We showed that hospital work was associated with increased risk of nasal *S. aureus* carriage. Repeated exposure to *S. aureus* in healthcare environments makes it likely that HW could be more frequently colonized. Previous studies have shown that there is high concordance between *S. aureus* strains isolated from medical staff and those from inpatients [24, 25]. In an international cross-sectional study, the prevalence of nasal *S. aureus* carriage in hospital workers was 39.4 %, and multivariate analysis showed that these subjects were at significantly higher risk of nasal *S. aureus* carriage regardless of their profession [26]. In contrast, in a population-based study by Olsen K et al. carried out in 2007–2008 in Norway, 26.2 % of healthcare workers and 26.0 % of non-healthcare workers showed *S. aureus* nasal carriage [27]. Healthcare worker status was not associated with *S. aureus* nasal carriage in the total population; however, the current study suggested that synergism between environmental risk factors (work and household) is important for *S. aureus* carrier status in HW [27]. In this study, we also showed that carriage rates were not significantly different between the CR (25.3 %) and HW (21.6 %) groups. Nevertheless, having family members in the healthcare profession was a significant risk factor for *S. aureus* nasal carriage in the HW group. *S. aureus* nasal carriers may ‘impose’ their carrier status upon other household members, and the bacterium can be reintroduced into the hospital by intra-familial spread to and from HW [26].

Higher *S. aureus* carriage rates have been associated with young age, being male, and underlying comorbidities [2–4, 13]. Interestingly, our study showed that independent risk factors for *S. aureus* carriage were different for CR and HW. The risk factors for the carriage of *S. aureus* among CR included being male, age ≤ 30 years, and nasal cavity cleaning habits. Our study provides important evidence that cleaning the nasal cavity with regularity might protect against nasal colonization by *S. aureus.* Cleaning the nasal cavity may change a microenvironment in the nose that protects against the growth of *S. aureus.* Published data showed that compliance with basic rules of hygiene, such as washing hand and using hydro-alcoholic solutions, could reduce the risk of nasal *S. aureus* colonization. Hydro-alcoholic solutions can interrupt auto-transmission of the pathogen, consequently decreasing the overall nasal carriage rates, especially in transient carriers [26]. Unfortunately, no further information about products or methods of cleaning the nasal cavity were collected in the current study. Clearly, the effect of cleaning the nasal cavity on *S. aureus* colonization requires further study. The other known risk factors could not be confirmed with significant associations in our study. Although the respective trends were visible, our study population most likely had insufficient power regarding less prevalent risk factors in the general population.

Previous studies from China have determined that the most common *S. aureus* strains, both nasal and clinical, belong to five major CCs: CC5, CC8, CC188, ST398, and CC59 [13, 21, 28, 29]. While the majority of the MSSA clones observed in China are globally distributed, ST7 and CC188 occurred at a relatively higher frequency in the recent Chinese studies [28, 30]. In our study, we found CC1, CC5, CC59, and CC188 *S. aureus* isolates were distributed between CR and HW. ST59, which is related to the CC59 clone, is the most frequent CA-MRSA clone, and most of the CA-MRSA strains causing infections in China belong to this ST [28, 31]. ST59/SCC*mec* V (PVL-positive) has become the most dominant CA-MRSA genotype in Taiwan, accounting for 69–84 % of CA-MRSA isolates in 1997–2005 [32]. ST59/SCC*mec* IV (PVL-negative) is the second-most dominant CA-MRSA genotype (accounting for 8.8–17.6 %), but it is the most prevalent type (accounting for 46–59 %) for nasal MRSA strains in the community [28]. Other detected CA-MRSA clones belong to ST338, ST45, ST910, and ST1349 [30, 31, 33]. However, in our study, only one ST59/SCC*mec* IVa strain was identified, and was isolated from a nurse working in the emergency department. Interestingly, the other three MRSA-SCC*mec* III strains belonged to ST8, ST10, and ST121, which are not common STs in China.

Also of note is the relatively high frequency (16.7 %) of HW isolates belonging to ST398. This genotype is frequently associated with animal infection, and individuals in close contact with animals are more likely to harbor ST398 isolates [29, 34]. However, we did not find any association between livestock contact and ST398 carriage in HW. Recently published data suggested that ST398 was the most prevalent clone among *S. aureus* isolates in healthy adults [13], and accounted for all *S. aureus* isolates associated with skin soft tissue infection in adults from Beijing [13, 29]. In our study, the three nurses carrying ST398 MSSA had direct contact with two patients with MRSA ST398/SCC*mec* III infection. These five ST398 isolates showed a high level of genetic similarity based on PFGE typing (data not shown). These findings may indicate that healthcare personnel could contribute to the dissemination of ST398 strains between hospitals and within the community, which should be investigated further.

The combined actions of many virulence factors enable *S. aureus* to cause disease [35]. Depending on these factors and on the immune status of the host, staphylococci can cause diseases ranging from superficial skin infections to deep-seated and systemic conditions such as osteomyelitis, septic shock, necrotizing pneumonia, and bromatoxism [36]. PVL has been intensely investigated because the two genes coding for the toxin (*lukS-PV* and *lukF-PV*) are the only virulence genes to have been epidemiologically linked to CA-MRSA infections [35]. However, although previous reports found an association between SCCmec IV-harboring clones and PVL production [28], none of the MRSA strains from our study tested positive for the PVL genes. However, *pvl* genes were present in 138 isolates with different genetic backgrounds. The diversity of *pvl*-positive strains might be attributed to the fact that the genes are localized on phages, which assist with the spread of the *pvl* genes through *S. aureus* populations [37].

*S. aureus* enterotoxins (SE) are a major cause of staphylococcal food poisoning [38]. SEA (and a combination of multiple superantigens) is associated with severe disease. SEB may suppress the motility of human polymorphonuclear neutrophils through the inhibition of exoprotein expression, and allow MRSA to invade and damage tissues [39]. Previous studies have identified SEA in isolates from patients with bacteremia and SEB in isolates from sputum samples, and confirmed that the corresponding genes are the most abundant toxin genes in clinical *S. aureus* isolates from patients and children in China [31, 40]. An important finding of the current study was the high rate of detection of the SE genes in *S. aureus* strains isolated from CR and HW. It is interesting to note that the prevalence of the *sea* gene was significantly higher in isolates from CR compared with those from HW, whereas the *seb* gene was more common among isolates from HW. This suggests that the toxin genes prevalent in *S. aureus* differ from the genes associated with other types of *S. aureus* infections.

Antimicrobial resistance in *S. aureus* has become an increasingly prevalent problem. *S. aureus* strains can differ in their susceptibility profiles, particularly between community- and healthcare-associated groups [41, 42]. In this study, antibiotic susceptibility tests revealed that most of the *S. aureus* isolates remained sensitive to the majority of antibiotics, but there was a high rate of resistance against penicillin and erythromycin. This resistance pattern might be the result of the excessive use of penicillin and macrolides. In comparison with previous reports [41, 42], the *S. aureus* isolates showed high levels of sensitivity to gentamicin, rifampicin, teicoplanin, quinupristin/dalfopristin, and trimethoprim/sulfamethoxazole. Therefore, this data should be considered for inclusion in epidemiologic datasets for both areas.

The current work has several limitations. Most importantly, because this was a cross-sectional study, it was not possible to detect variations in colonization patterns, e.g. persistent carriers, intermittent carriers, or non-carriers. Secondly, there are some differences in the demographic profiles of the CR and HW groups, including significantly different age and gender distributions. Although selection of participants was random at Sun Yat-Sen Memorial Hospital, the age distribution among HW was 20–50 years, and the majority of the volunteers were women (64.4 %, 188/292). In the present study, age and sex were not associated with nasal *S. aureus* carriage in the HW group. In contrast, recently published data suggest that high nasal carriage rates were found among nurses and respiratory and laboratory technicians [24, 26, 27]. In addition, recruitment of subjects from the university campus may mean that the results are not generalizable to the population of Guangzhou as a whole, despite the good cross sections of age and occupation among the test subjects. Thirdly, sampling only the nostrils without including other body parts may underestimate the frequency of MRSA carriage overall [42]. Finally, *spa* typing was not performed in the current study, and this is something that should be conducted in future analyses.

## Conclusions

These data indicate a low prevalence of nasal MRSA carriage but obvious evidence of molecular heterogeneity of *S. aureus* isolates between CR and HW at Sun Yat-Sen University, Guangzhou. Differences in epidemiological and molecular characteristics between populations may be useful for understanding *S. aureus* nasal colonization dynamics, and for designing strategies to prevent *S. aureus* infection and dissemination.

## Additional files

Additional file 1:
**STROBE-Checklist.** (PDF 189 kb) Additional file 2: Table S1. The primers used for *S. aureus* molecular typing with Multiplex PCR. **Table S2** Antimicrobial susceptibility profiles of *S. aureus* nasal isolates among CR and HW. **Table S3** Distribution of MLST, PFGE, *pvl, sea*, and *seb* within each *S. aureus* clonal complex (CC) detected in CR and HW at Sun Yat-Sen University, Guangzhou, China. (PDF 175 kb) Additional file 3: Figure S2. Genetic relatedness among *S. aureus* isolates from healthcare workers and community residents. Dendrogram based on PFGE SmaI restriction pattern analysis of 129 nares-colonizing *S. aureus* isolates. Similarity analysis was performed with Dice’s coefficient, and clustering was done by using the unweighted-pair group method using average linkages (UPGMA) method. The scale at the top shows percentages of similarity. Further information is shown on the right, including the antibiotics, key, sex, age and profession and PFGE types. Antimicrobial susceptibility tests (AST): black indicates resistance, grey indicates intermediate, and white indicates susceptibility. Abbreviations are as follows: FOX, cefoxitin; PEN, penicillin; CXM, cefuroxime; IPM, imipenem; CTX, cefotaxime; CRO, ceftriaxone; GEN, gentamicin; CIP, ciprofloxacin; ERY, erythromycin; VAN, vancomycin; RIF, rifampicin; TCY, tetracycline; TEC, teicoplanin; LVF, levofloxacin; CLI, clindamycin; SXT, trimethoprim/sulfamethoxazole; QD, quinupristin/dalfopristin; UG, Undergraduate; HW, Healthcare worker; MSS, Middle school student; SC, Salesclerk; RE, Retiree. (PDF 243 kb) Additional file 4: Figure S1. Detection of SCC*mec* types and virulence factor genes. A. Agarose gel electrophoresis showing the products amplified by SCC*mec* multiplex PCR. B-D. Agarose gel electrophoresis showing the PCR products amplified by Panton-Valentine Leucocidine (*pvl*), *Staphylococcus aureus* enterotoxins A (*sea*) and *Staphylococcus aureus* enterotoxins B (*seb*). (PDF 338 kb)

## Abbreviations

CC: Clonal complex
CI: Confidence interval
CR: Community residents
HW: Healthcare workers
MDR: Multidrug-resistant
MLST: Multilocus sequence typing
MRSA: Methicillin-resistant *S. aureus*
MSSA: Methicillin-sensitive *S. aureus*
SCC*mec*: Staphyloccoccal cassette chromosome *mec*
SE: *S. aureus* enterotoxins
ST: Sequence type
PFGE: Pulsed-field gel electrophoresis
PVL: Panton-Valentine Leukocidin
