# Supplementary material for: Differences in Staphylococcus aureus nasal carriage and molecular characteristics among community residents and healthcare workers at Sun Yat-Sen University, Guangzhou, Southern China
Source: BMC Infect Dis. 2015 Jul 30;15:303. doi: 10.1186/s12879-015-1032-7 (PMC4520063; doi:10.1186/s12879-015-1032-7)
Supplement: Additional file 1: — STROBE-Checklist. (PDF 189 kb) [file 12879_2015_1032_MOESM1_ESM.pdf]

STROBE Statement—checklist of items that should be included in reports of observational studies

	Item No	Recommendation
**Title and abstract**	1	(a) Indicate the study's design with a commonly used term in the title or the abstract （Page 1）
		(*b*) Provide in the abstract an informative and balanced summary of what was done and what was found (Page 3-4)
**Introduction**		
Background/rationale	2	Explain the scientific background and rationale for the investigation being reported (Page 4-6)
Objectives	3	State specific objectives, including any prespecified hypotheses (Page 6)
**Methods**		
Study design	4	Present key elements of study design early in the paper (Page 6-8)
Setting	5	Describe the setting, locations, and relevant dates, including periods of recruitment, exposure, follow-up, and data collection (Page 6-7)
Participants	6	*Cross-sectional study*—Give the eligibility criteria, and the sources and methods of selection of participants (Page 7)
Variables	7	Clearly define all outcomes, exposures, predictors, potential confounders, and effect modifiers. Give diagnostic criteria, if applicable (Page 7-8)
Data sources/ measurement	8*	For each variable of interest, give sources of data and details of methods of assessment (measurement). Describe comparability of assessment methods if there is more than one group (Page 12)
Bias	9	Describe any efforts to address potential sources of bias (Page 8-10)
Study size	10	Explain how the study size was arrived at (Page 7)
Quantitative variables	11	Explain how quantitative variables were handled in the analyses. If applicable, describe which groupings were chosen and why (Page 12-13)
Statistical methods	12	(*a*) Describe all statistical methods, including those used to control for confounding (Page 10-12)
		(*b*) Describe any methods used to examine subgroups and interactions (Page 10-12)
		(*c*) Explain how missing data were addressed (Page 7-8)
		(*d*) *Cross-sectional study*—If applicable, describe analytical methods taking account of sampling strategy (Page 6-8)
		(*e*) Describe any sensitivity analyses (Page 8-10)

Continued on next page

**Results**		
Participants	13*	(a) Report numbers of individuals at each stage of study—eg numbers potentially eligible, examined for eligibility, confirmed eligible, included in the study, completing follow-up, and analysed<br>(Page 7,12-13)
		(b) Give reasons for non-participation at each stage<br>(Page 7)
		(c) Consider use of a flow diagram<br>No use (as the experiment process could be reflected from the experimental method, so the flow diagram was not used)
Descriptive data	14*	(a) Give characteristics of study participants (eg demographic, clinical, social) and information on exposures and potential confounders<br>(Page 12-13,31-33)
		(b) Indicate number of participants with missing data for each variable of interest<br>(Page 31-33)
Outcome data	15*	*Cross-sectional study*—Report numbers of outcome events or summary measures<br>(Page 12-17)
Main results	16	(*a*) Give unadjusted estimates and, if applicable, confounder-adjusted estimates and their precision (eg, 95% confidence interval). Make clear which confounders were adjusted for and why they were included<br>(Page 12-17, 31-33)
		(*b*) Report category boundaries when continuous variables were categorized<br>(Page 12-13, 32-33)
		(*c*) If relevant, consider translating estimates of relative risk into absolute risk for a meaningful time period<br>(Page 12-13, 32-33)
Other analyses	17	Report other analyses done—eg analyses of subgroups and interactions, and sensitivity analyses<br>(Page 12-17, 32)
**Discussion**		
Key results	18	Summarise key results with reference to study objectives<br>(Page 17-25)
Limitations	19	Discuss limitations of the study, taking into account sources of potential bias or imprecision. Discuss both direction and magnitude of any potential bias<br>(Page 20, 24-25)
Interpretation	20	Give a cautious overall interpretation of results considering objectives, limitations, multiplicity of analyses, results from similar studies, and other relevant evidence<br>(Page 18-24)
Generalisability	21	Discuss the generalisability (external validity) of the study results<br>(Page 20,22-25)
**Other information**		
Funding	22	Give the source of funding and the role of the funders for the present study and, if applicable, for the original study on which the present article is based<br>(Page 26)

*Give information separately for cases and controls in case-control studies and, if applicable, for exposed and unexposed groups in cohort and cross-sectional studies.

Note: An Explanation and Elaboration article discusses each checklist item and gives methodological background and published examples of transparent reporting. The STROBE checklist is best used in conjunction with this article (freely available on the Web sites of PLoS Medicine at http://www.plosmedicine.org/, Annals of Internal Medicine at http://www.annals.org/, and Epidemiology at http://www.epidem.com/). Information on the STROBE Initiative is available at www.strobe-statement.org.
