# Supplementary material for: Differences in Staphylococcus aureus nasal carriage and molecular characteristics among community residents and healthcare workers at Sun Yat-Sen University, Guangzhou, Southern China
Source: BMC Infect Dis. 2015 Jul 30;15:303. doi: 10.1186/s12879-015-1032-7 (PMC4520063; doi:10.1186/s12879-015-1032-7)
Supplement: Additional file 2: Table S1. — The primers used for S. aureus molecular typing with Multiplex PCR. Table S2 Antimicrobial susceptibility profiles of S. aureus nasal isolates among CR and HW. Table S3 Distribution of MLST, PFGE, pvl, sea, and seb within each S. aureus clonal complex (CC) detected in CR and HW at Sun Yat-Sen University, Guangzhou, China. (PDF 175 kb) [file 12879_2015_1032_MOESM2_ESM.pdf]

Table S1 The primers used for *S. aureus* molecular typing with Multiplex PCR.

	Primers	Primer sequence（5'-3'）	Products size（bp）
PVL gene	Luk-PV	F：ATCATTAGGTAAAATGTCTGGACATGATCCA	
		R：GCATCAAGTGTATTGGATAGCAAAAGC	433bp
SCC*mec* typing	Type I	F：GCTTTAAAGAGTGTCGTTACAGG	
		R：GTTCTCTCATAGTATGACGTCC	613bp
	Type II	F：CGTTGAAGATGATGAAGCG	
		R：CGAAATCAATGGTTAATGGACC	398bp
	Type III	F：CCATATTGTGTACGATGCG	
		R：CCTTAGTTGTCGTAACAGATCG	280bp
	Type IVa	F：GCCTTATTCGAAGAAACCG	
		R：CTACTCTTCTGAAAAGCGTCG	776bp
	Type IVb	F：TCTGGAATTACTTCAGCTGC	
		R：AAACAATATTGCTCTCCCTC	493bp
	Type IVc	F：ACAATATTTGTATTATCGGAGAGC	
		R：TTGGTATGAGGTATTGCTGG	200bp
	Type IVd	F：CTCAAAATACGGACCCCAATACA	
		R：TGCTCCAGTAATTGCTAAAG	881bp
	Type V	F：GAACATTGTTACTTAAATGAGCG	
		R：TGAAAGTTGTACCCTTGACACC	325bp
MLST typing	arcc	F： TTGATTCACCAGCGCGTATTGTC	
		R： AGGTATCTGCTTCAATCAGCG	456bp
	aroe	F：ATCGGAAATCCTATTTCACATTC	
		R：GGTGTTGTATTAATAACGATATC	456bp
	glpf	F：CTAGGAACTGCAATCTTAATCC	
		R：TGGTAAAATCGCATGTCCAATTC	465bp
	gmk	F：ATCGTTTTATCGGGACCATC	
		R：TCATTAACTACAACGTAATCGTA	429bp
	pta	F：GTTAAAATCGTATTACCTGAAGG	
		R：GACCCTTTTGTTGAAAAGCTTAA	474bp
	tpi	F：TCGTTCATTCTGAACGTCGTGAA	
		R：TTTGCACCTTCTAACAATTGTAC	402bp
	yqil	F：CAGCATACAGGACACCTATTGGC	
		R：CGTTGAGGAATCGATACTGGAAC	516bp
SEA genes	*sea*	F：CTGAATTGCAGGGAACAGCT	
		R： CGTCTTGCTTGAAGATCCAACT	436bp
SEB genes	*seb*	F：AATCTATAGATCAATTTCTATAC	
		R：CTTTTTCTTTGTCGTAAGATA	602bp

Table S2 Antimicrobial susceptibility profiles of *S. aureus* nasal isolates among CR and HW.

Antimicrobial agent	Total (n=138)	CR (n=75)			HW (n=63)			P-value
	R (%)	R (%)	I (%)	S (%)	R (%)	I (%)	S (%)	
Penicillin	82.5	83.6	0	16.4	81.2	0	18.8	0.581
Erythromycin	39.5	43.8	5.5	50.7	34.4	4.7	60.9	0.323
Clindamycin	14.6	13.7	13.7	72.6	15.6	9.4	75	0.542
Cefuroxime	2.9	1.4	0	98.6	4.7	0	95.3	0.106
Ceftriaxone	4.4	4.1	0	95.9	4.7	1.6	93.7	0.089
Cefotaxime	4.4	4.1	0	95.9	4.7	0	95.3	0.089
Cefoxitin	2.9	1.4	0	98.6	4.7	0	95.3	0.106
Gentamicin	9.5	9.6	1.4	89	9.4	1.6	89	0.821
Imipenem	2.9	1.4	0	98.6	4.7	0	95.3	0.106
Ciprofloxacin	2.1	0	6.8	93.2	4.7	17.2	78.1	<0.001
Levofloxacin	2.1	0	0	100	4.7	1.6	93.8	<0.001
Rifampicin	3.6	4.1	0	95.9	3.1	0	96.9	0.612
Trimethoprim/sulfamethoxazole	6.6	5.5	0	94.5	7.8	0	92.2	0.578
Tetracycline	18.9	17.8	0	82.2	20.3	0	79.7	0.724
Teicoplanin	0	0	0	100	0	0	100	
Quinupristin/dalfopristin	0	0	0	100	0	0	100	
Vancomycin	0	0	0	100	0	0	100	

R, resistance; I, intermediate; S, susceptibility.

No resistance was detected in both areas for Tetracycline, Quinupristin/dalfopristin and Vancomycin.

Table S3 Distribution of MLST, PFGE, *pvl*, *sea*, and *seb* within each *S. aureus* clonal complex (CC) detected in CR and HW at Sun Yat-Sen University, Guangzhou, Southern China.

CC	n	MLST		PFGE		SCC*mec* (n=4)	*pvl* (n=2)	*sea* (+)*seb*(-) (n=7)	*sea* (-)*seb*(+) (n=8)	*sea*(+)*seb*(+) (n=23)	*sea*(-)*seb* (-) (n=3)
		CR (n=23)	HW (n=18)	CR (n=23)	HW (n=18)						
CC1	5	1(3),2139(1)	432(1)	A(3),J(1)	A(1)	-	0/5	2/5	0/5	3/5	0/5
CC5	5	5(1),965(2)	5(2)	A(3)	I(2)	-	1/5	0/5	0/5	4/5	1/5
CC6	3	6(2)	6(1)	A(2)	A(1)	-	1/3	2/3	1/3	0/3	0/3
CC7	2	7(2)	-	V(1)	-	-	0/2	0/2	0/2	2/2	0/2
CC8	2	8(1)	630(1)	D(1),-	C(1)	III	0/2	0/2	1/2	1/2	0/2
CC10	1	-	10(1)	-	G(1)	III	0/1	0/1	0/1	0/1	1/1
CC15	1	-	15(2)	-	E(2)	-	0/2	0/2	0/2	2/2	0/2
CC30	2	30(2)	-	N(2)	-	-	0/2	1/2	0/2	1/2	0/2
CC59	6	59(4),537(1)	59(1)	L(4),M(1)	C(1)	IVa	0/2	2/6	1/6	3/6	0/6
CC72	1	72(1)	-	A(1)	-	-	0/1	0/1	0/1	1/1	0/1
CC121	1	-	120(1)	-	T(1)	III	0/1	0/1	0/1	1/1	0/1
CC182	1	944(1)	-	S(1)	-	-	0/1	0/1	0/1	1/1	0/1
CC188	7	188(3), 3073(1)	188(3)	A(4)	O(2),A(1)	-	0/7	0/7	5/7	1/7	1/7
CC398	3	-	398(3)	-	A(3)	-	0/3	0/3	0/3	3/3	0/3

CR, community residents; HW, healthcare workers; -, not detected
