# Supplementary figures and images for: Differences in Staphylococcus aureus nasal carriage and molecular characteristics among community residents and healthcare workers at Sun Yat-Sen University, Guangzhou, Southern China
Source: BMC Infect Dis. 2015 Jul 30;15:303. doi: 10.1186/s12879-015-1032-7 (PMC4520063; doi:10.1186/s12879-015-1032-7)

Figure S2

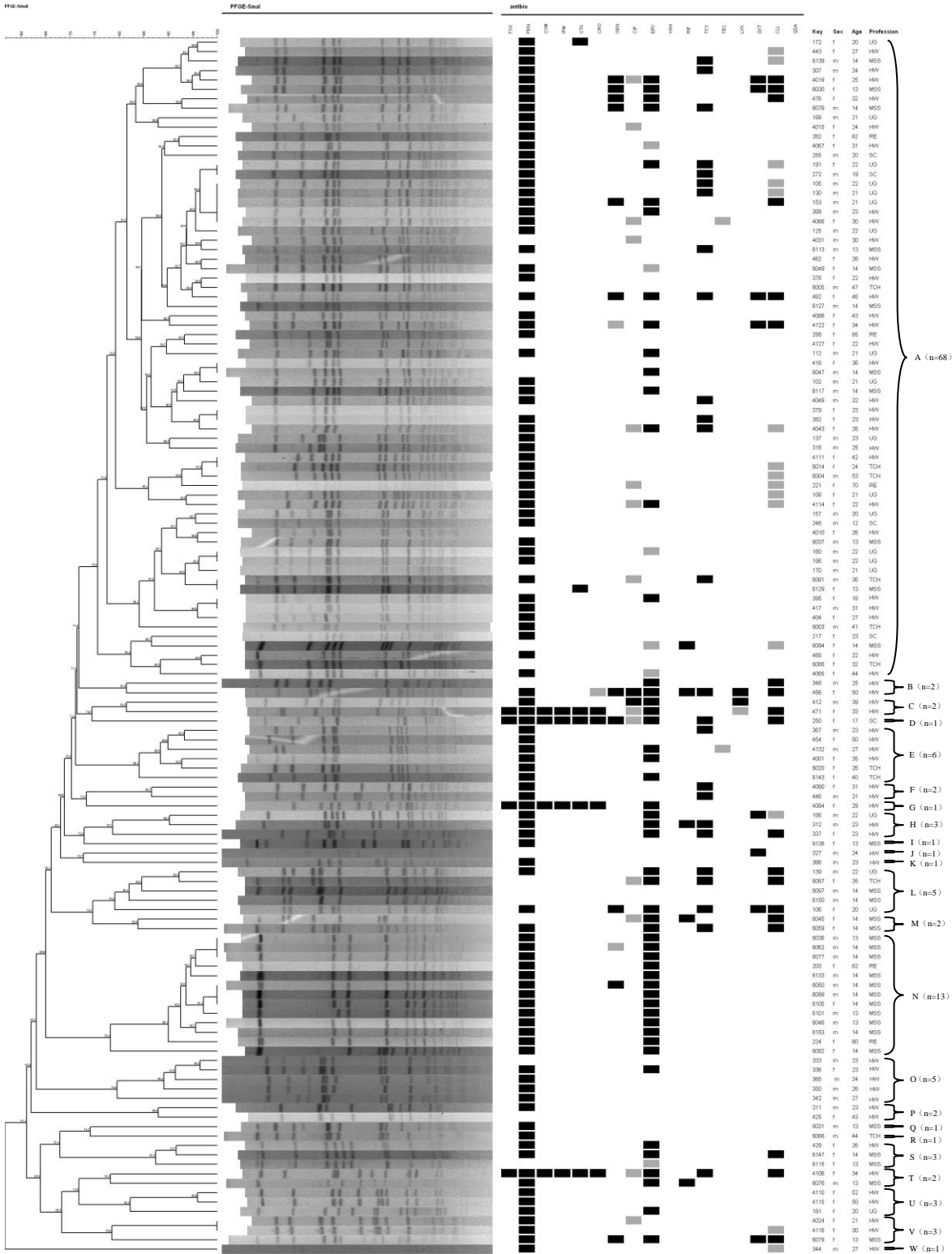

Supplement: Additional file 3: Figure S2. — Genetic relatedness among S. aureus isolates from healthcare workers and community residents. Dendrogram based on PFGE SmaI restriction pattern analysis of 129 nares-colonizing S. aureus isolates. Similarity analysis was performed with Dice’s coefficient, and clustering was done by using the unweighted-pair group method using average linkages (UPGMA) method. The scale at the top shows percentages of similarity. Further information is shown on the right, including the antibiotics, key, sex, age and profession and PFGE types. Antimicrobial susceptibility tests (AST): black indicates resistance, grey indicates intermediate, and white indicates susceptibility. Abbreviations are as follows: FOX, cefoxitin; PEN, penicillin; CXM, cefuroxime; IPM, imipenem; CTX, cefotaxime; CRO, ceftriaxone; GEN, gentamicin; CIP, ciprofloxacin; ERY, erythromycin; VAN, vancomycin; RIF, rifampicin; TCY, tetracycline; TEC, teicoplanin; LVF, levofloxacin; CLI, clindamycin; SXT, trimethoprim/sulfamethoxazole; QD, quinupristin/dalfopristin; UG, Undergraduate; HW, Healthcare worker; MSS, Middle school student; SC, Salesclerk; RE, Retiree. (PDF 243 kb) [file 12879_2015_1032_MOESM3_ESM.pdf]

# Figure S1

## A. SCCmec typing

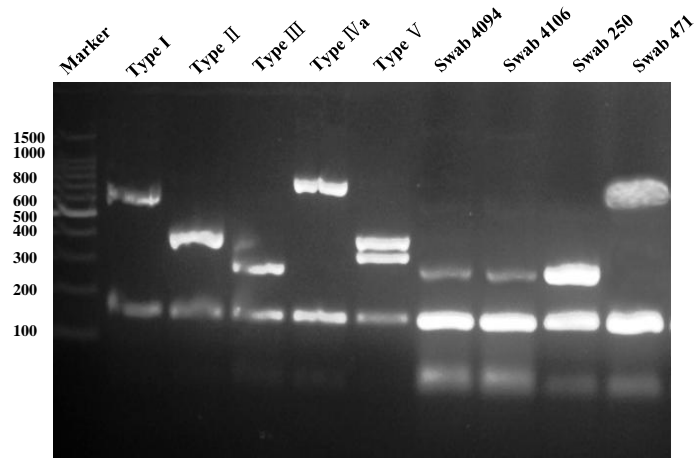

## C. *sea*

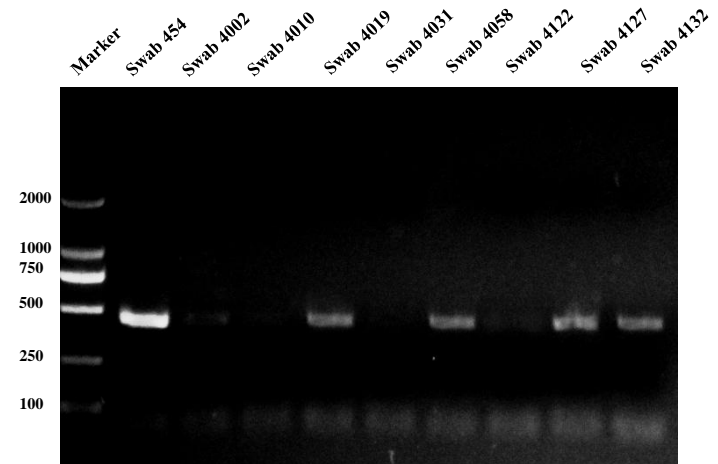

## B. *pvl*

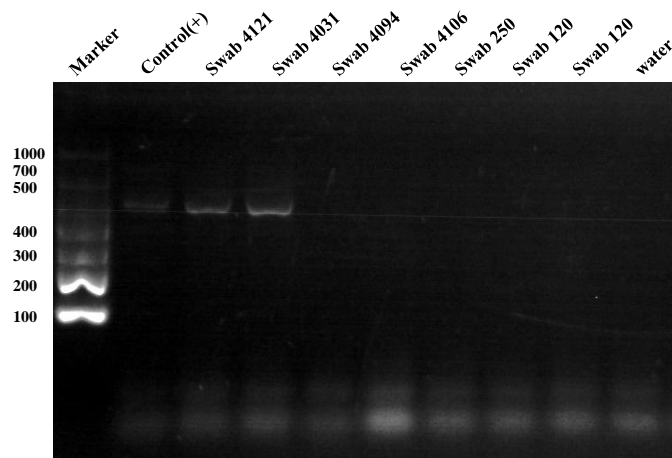

## D. *seb*

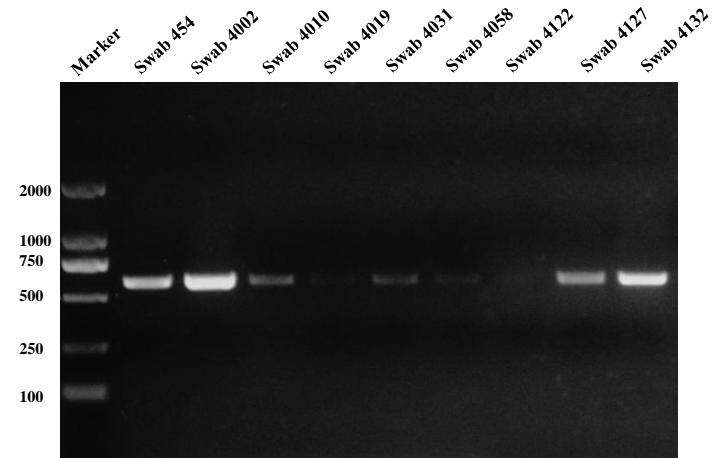

Supplement: Additional file 4: Figure S1. — Detection of SCCmec types and virulence factor genes. A. Agarose gel electrophoresis showing the products amplified by SCCmec multiplex PCR. B-D. Agarose gel electrophoresis showing the PCR products amplified by Panton-Valentine Leucocidine (pvl), Staphylococcus aureus enterotoxins A (sea) and Staphylococcus aureus enterotoxins B (seb). (PDF 338 kb) [file 12879_2015_1032_MOESM4_ESM.pdf]
